# Transport Infrastructure Shapes Foraging Habitat in a Raptor Community

**DOI:** 10.1371/journal.pone.0118604

**Published:** 2015-03-18

**Authors:** Aimara Planillo, Stephanie Kramer-Schadt, Juan E. Malo

**Affiliations:** 1 Terrestrial Ecology Group (TEG), Departamento de Ecología, Universidad Autónoma de Madrid, Madrid, Spain; 2 Department of Evolutionary Ecology, Leibniz Institute for Zoo and Wildlife Research, Berlin, Germany; University of Lleida, SPAIN

## Abstract

Transport infrastructure elements are widespread and increasing in size and length in many countries, with the subsequent alteration of landscapes and wildlife communities. Nonetheless, their effects on habitat selection by raptors are still poorly understood. In this paper, we analyzed raptors’ foraging habitat selection in response to conventional roads and high capacity motorways at the landscape scale, and compared their effects with those of other variables, such as habitat structure, food availability, and presence of potential interspecific competitors. We also analyzed whether the raptors’ response towards infrastructure depends on the spatial scale of observation, comparing the attraction or avoidance behavior of the species at the landscape scale with the response of individuals observed in the proximity of the infrastructure. Based on ecological hypotheses for foraging habitat selection, we built generalized linear mixed models, selected the best models according to Akaike Information Criterion and assessed variable importance by Akaike weights. At the community level, the traffic volume was the most relevant variable in the landscape for foraging habitat selection. Abundance, richness, and diversity values reached their maximum at medium traffic volumes and decreased at highest traffic volumes. Individual species showed different degrees of tolerance toward traffic, from higher abundance in areas with high traffic values to avoidance of it. Medium-sized opportunistic raptors increased their abundance near the traffic infrastructures, large scavenger raptors avoided areas with higher traffic values, and other species showed no direct response to traffic but to the presence of prey. Finally, our cross-scale analysis revealed that the effect of transport infrastructures on the behavior of some species might be detectable only at a broad scale. Also, food availability may attract raptor species to risky areas such as motorways.

## Introduction

The impact of human modified landscapes on wildlife is an important but yet not well understood issue. Human disturbances change community composition and modify ecosystems [1–3]. Among human alterations, elements of the transport infrastructure (i.e. roads) are a common feature in many countries, and they are likely to increase in number, extent and intensity of use all over the world in the near future. Transport infrastructure alters the surrounding environment with varying effects on vertebrate species [4, 5]. Species may be affected positively by the creation of new habitat or corridors [6–8], or negatively by direct mortality, fragmentation, noise disturbance, or habitat loss [9–11]. Also the extent of these effects is not restricted to areas beside roads and can spread up to several kilometers for large species [12, 13].

Many studies have focused on the effect of roads on terrestrial vertebrates, but few of them were centered in flying species [5, 12]. Nonetheless, transport infrastructure can pose a serious threat to many birds. Roads might seem attractive to some species as they can provide valuable resources for birds like perches, food, or nesting sites in shrubs in their verges or adjacent structures [14, 15]. However, birds close to roads suffer negative population effects such as decreased breeding success or direct mortality by vehicle collision [16–18]. In general, the more mobile the bird species, the higher the road impacts [19].

In this context, the response of diurnal raptors to roads is of special interest. Most raptor species are listed under several categories on the IUCN red list [20], they are targets of conservation programs, and they also have an important ecological role in ecosystems as top predators. Moreover, habitat alterations are among the main factors behind the decline of raptor populations over the last years [21–23]. Transport infrastructure effects have been studied at local scales, with some species selecting roads due to food or perching site availability [24–26]. However, traffic volume might change the response of raptors to roads, especially large species, decreasing road use of some of them when traffic increases [26]. Few studies have analyzed the habitat selection of raptors at broader spatial scales, and raptor community response to transport infrastructure at the landscape scale remains unclear. Contrary to local scale studies, Knight and Kawashima [27] failed to find differences between roads and control sites for red-tailed hawks (*Buteo jamaicensis*), and scavenger raptors showed different responses depending on the species [28].

Species can select habitat at different scales, from landscape to local scale. Although sometimes different scales can share important variables [29], it is expected that the main response of the species to habitat features occurs at the landscape scale, according to the hierarchical habitat selection (HHS) hypothesis [13, 30].Thus, to understand raptor habitat selection, we first need to understand the important factors at the landscape scale. Accordingly, most studies have focused on the effects of factors at this scale related to habitat, food availability, competition, or even human activities [31–33]. However, two interesting questions remain unexplored: how important are road effects in comparison to other factors for raptors’ habitat selection, and whether the raptors’ response to roads detected at the local scale is comparable to that at the broad scale.

In order to disentangle the effects of the transport infrastructure on raptor community, we studied the foraging habitat selection of diurnal raptors during their daily movements. We analyzed raptors’ responses to roads with different levels of traffic, including also other variables linked to habitat selection such as habitat structure, food availability and interactions with other potentially competing species. Our objectives were to identify the most relevant variables for the foraging habitat selection of raptors at landscape scale and to compare the response of raptors to traffic detected at a landscape scale with that observed at a local scale. Based on the existing literature for habitat selection and road effects on raptors, our hypotheses were: 1. Transport infrastructures will be among factors affecting foraging habitat selection of raptors at landscape scale, as it is been shown for some species at local scales [24–26]. We predict that the presence of roads will affect raptor behavior, with larger effects for higher traffic levels, due to the disturbing effect of traffic [4]. 2. The change in the raptor community is expected to occur both in species composition and abundance [34, 35]. We predict that the most tolerant and opportunistic species will be more abundant near infrastructures, whereas the less tolerant will avoid them despite the availability of resources. 3. Those species strongly affected by transport infrastructures will show similar responses to traffic across scales [30]. We predict that only individuals from opportunistic tolerant species will select transport infrastructures at both scales, while less tolerant species will avoid them at least at one of the scales to minimize the perturbation of the infrastructure [13].

## Materials and Methods

### Study Area

**Fig 1:**
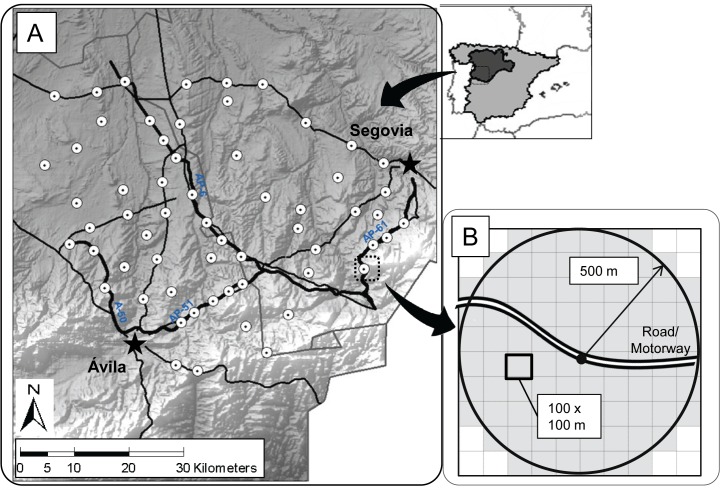
Study area and survey design. A. Location of the study area and the observation points (white dots) in Castilla y León Region, Central Spain. Thin black lines represent 2 lane roads and bold black lines represent motorways. Main cities in the area are displayed with a star. Base map downloaded from Instituto Geográfico Nacional de España (IGN, http://www.ign.es). B. Detail of a sampling point either in a road or motorway, and the grid used for local scale analyses (shaded area) of 100 x 100 m cells inside the 500 m radius.

The study was carried out in an area of 3600 km^2^ in southern Castilla y León Region, Central Spain (Fig. 1A). It is a rural area, with an average population density of 25.5 inhabitants per km^2^. The climate is continental Mediterranean, with cold winters and dry summers, and average annual precipitation about 490 mm. The landscape can be categorized into three main formations: agricultural lands of non-irrigated cereals, pastures with oak trees used for cattle grazing (“dehesas”), and patches of natural vegetation, characterized by holm oak forests (*Quercus ilex*) and Mediterranean shrub formations. Areas of high conservation value included in the European Natura 2000 Network extend over 500 km^2^ in the study area.

Four motorways cross the area, characterized by 4 to 6 traffic lanes and a speed limit of 120 kmh^−1^. Motorways can be divided into medium-traffic motorways (AP-51, AP-61 and A-50), with an average daily traffic (ADT) of 6,500–8,000 vehicles and an approximate length inside the study area of 80 km, and high-traffic motorways (AP-6), with an ADT of 20,000 vehicles and an approximate length of 65 km. In addition, there are several 2-lane roads connecting small cities and villages, with speed limits of 90–100 kmh^−1^. Roads usually support less traffic volume and their ADT ranged from 1,000 to 3,300 vehicles (with some punctual busy stretches up to 6,000 vehicles). AP-51, AP-61 and AP-6 motorways belong to ABERTIS-IBERPISTAS that granted permission to work on them.

### Survey design and data collection

Data on raptor foraging habitat selection was taken on 60 sampling plots distributed throughout the study area, 20 along motorways, 20 along main two-lane roads and 20 in control sites. We consider control sites as those that were at least 3 km away from roads and motorways. Sampling plots were randomly distributed, in places with good visibility, always next to the asphalt surface on roads and motorways, and at least 3 km from the nearest sampling plot to ensure independence of the observations (Fig. 1A). Each sampling plot was visited 4 times, two times in the breeding season (June 2010 and 2011), and two times in the winter season (January-February 2011 and 2012).

The sampling plot was defined as the area in a radius of 500 m from the observer’s position, with road and motorway plots always centered on the infrastructure. During each visit, one observer recorded all the individuals showing foraging behavior inside the sampling plot during 30 minutes. We assumed raptors to be foraging when they were actively hunting or searching the ground by soaring at low altitude. Surveys were conducted during the whole day, three plots during morning and three during afternoon, one from each zone and alternating the order of the zones from one day to the next to avoid hour bias. To avoid sampling biases due to weather dependent factors [36], or to changes in human disturbances on weekends [26], the field work was restricted to windless and rainless work days starting 2–3 hours after sunrise and finishing two hours before sunset.

### Variables used for landscape scale analysis

At a landscape scale, we analyzed the foraging habitat selection of raptors both at community and species-specific levels, with special focus on the traffic effects.

Response variables. Individuals were identified at species level whenever possible. For the community level responses, the number of individuals detected per plot in each survey was taken as the relative abundance and the number of species as the relative richness. We also estimated the Shannon-Wiener diversity index for each plot. For the species level response, we analyzed the abundance of the species with more than 40 observations: red kite (*Milvus milvus*), black kite (*Milvus migrans*), booted eagle (*Hieraaetus pennatus*), common buzzard (*Buteo buteo*), griffon vulture (*Gyps fulvus*), cinereous vulture (*Aegypius monachus*) and kestrels (S1 Table). During the breeding season two kestrel species (*Falco tinnunculus* and *Falco naumanni*) share the area and their determination at the species level was not always certain. Therefore both species were pooled together.

Predictor variables. For each sampling plot, we estimated seven predictor variables based on literature for foraging habitat selection, and that were related to the infrastructure (see the list and a brief description in Table 1).

**Table 1:** Predictor variables for the landscape scale analysis.

Code	Predictor Description	N	Values (mean ± SE)
ADT	Average daily traffic. Obtained each natural year as an estimate of the number of vehicles a day that drive through each sampling plot. Continuous variable.	180	4888 ± 371.47 (0–20850)
Habitat	Main vegetation of each sampling point. Factor.	60	Crops, Monte^1^, Pines, Unproductive, Mixed^2^.
D.vill	Distance to the nearest village. Continuous variable, Log-transformed.	60	3.11 ± 0.04 (2.19–3.83)
Rabbits	Relative rabbit abundance. Sum of rabbit pellets in 10 plots of 0.5 m^2^, evenly spaced along a 1km transect inside the sampling plot. Continuous variable, Log-transformed. Obtained during breeding season.	120	1.03 ± 0.06 (0–2.58)
Micros	Relative small mammal abundance. Sum of warrens found in 10 plots of 5 m radius, evenly spaced along a 1km transect inside the sampling plot. Counts. Obtained during breeding season.	120	14.13 ± 1.14 (0–126)
HT.Rkill^a^	Distance to the nearest roadkill hotspot in high-traffic motorway. Continuous variable, Log-transformed.	120	3.88 ± 0.11 (0.92–4.44)
MT.Rkill^a^	Distance to the nearest roadkill hotspot in medium-traffic motorways. Continuous variable,Log-transformed.	120	4.15 ± 0.05 (2.66–4.67)
season	Controlling variable. Season of each survey. Factor.	240	Breeding, Winter
time	Controlling variable. Time of the day of the survey. Factor.	240	Morning, Afternoon
visib	Controlling variable. Proportion of the sampling plot fully visible due to terrain constrains. Proportion.	60	0.92 ± 0.01 (.56–1)

For each predictor there is a code used in the statistical analysis and a brief description. Values are presented as mean ± SE and range for quantitative variables, and levels for categorical factors.

^a^Distances to roadkill hotspots were calculated in winter and breeding season independently, based on roadkill database and two year roadkill monitoring (unpublished data).

^1^ Holm oak forest and dehesas with high shrub cover.

^2^ Sampling points with no clear dominance of any vegetation classes.

The sampling plots belonged to one of the three categories of traffic infrastructure types used for the stratification of the survey design. The stratification carried out for sampling in control, road and motorway sites reflects a parallel gradient in two underlying variables: traffic volume and speed limit. Thus, the highest traffic volumes occur in motorways, which also have the highest speed limit (120 kmh^−1^). The opposite situation appears in control sites (no traffic and ‘0’ speed limit), while roads have intermediate traffic levels with speed limits around 90 kmh^−1^ (as we only used main roads for our study). Since traffic volume has been shown to have an effect on the response of some raptor species to roads [26], we decided to use traffic volume, measured as average daily traffic (ADT), to test the general effect of transport infrastructure. We assigned each sampling plot to a habitat type (see Table 1 for details), since some species have shown preference for certain habitats, for example, kestrels usually select open areas [23], common buzzards prefer forested areas [32], and black kites avoid woodland [23, 37]. The distribution of habitat types did not show significant differences among the survey traffic strata (χ2 = 8.55, p = 0.359 computed with a 2000 replicate Monte Carlo simulation), so we discarded a possible confounding effect of the habitat (e.g. one habitat type being more abundant in control sites than in motorways). We also calculated the distance to the nearest village because the proximity of urban areas can alter the habitat use of the species, for example black kites, booted eagles and kestrels have shown preference for villages due to availability of anthropogenic resources like garbage or anthropophilic prey, discards from farms and nesting sites in old buildings [33, 37–39], while common buzzards show avoidance of villages, probably due to excessive disturbance [33].

Road verges can act as refuges for small mammals and increase their abundance [6, 8]. Once a year, we estimated relative abundance indices of two main preys: small mammals (mainly voles) and rabbits. This survey was carried out during breeding season, as it is the productivity peak for those species. Both taxa are common prey for red and black kites, buzzards, and booted eagles [39–41]. Voles are also the main prey for common kestrels [38], and rabbit carcasses can be important in the diet of black vultures, and also consumed by griffon vultures [23]. Vultures are obligate scavengers, although other species in the area also use carrion, such as kites and buzzards [23].To test the effect of the availability of carrion, we identified roadkill hotspots in motorways following the method in Malo et al. [42]. This method detects clusters of animal collisions along a road and defines hotspots as those stretches containing a higher number of roadkills than would be expected from a random distribution (poisson distribution with the observed mean). Data on location of roadkills were obtained from a database provided by the motorway management agency and from a monthly survey carried out during two years. The total roadkill data showed very few ungulate casualties, with rabbits being the most common roadkilled species (unpublished data). Since the traffic density might affect the behavior of some raptors [26], we divided roadkill hotspots into two categories: those affected by medium traffic and those affected by high traffic volume. Distances to both types of hotspots were included in the analyses as separate variables to account for different responses to carrion depending on the traffic density.

We also measured three variables that could influence the flying behavior of raptors and their detectability, and included them in the statistical models when necessary: time of day, visibility and season (controlling variables in Table 1). Season was only included for those species that were present in the study area all year round (S1 Table).

All distance measurements and habitat assessment were done in ArcGIS 9.3, using public cartography and aerial photography from Castilla y León Region [43].

### Estimation of infrastructure use index

Within road and motorway sampling plots, we analyzed the response of raptors to the infrastructure itself against surrounding habitat (hereafter denoted as “infrastructure use”) to compare it with the response of the species to the infrastructure at the landscape scale. We superimposed a 100 x 100 grid to the plot (500 m radius) (Fig. 1B), and we defined an infrastructure use index based on the proportion of sightings for each individual located on asphalt cells related to the total amount of sightings for that individual. Total sightings per individual ranged from 1 to 6, depending on the time the raptor spent inside the sampling plot.

Cells of the grid traversed by the transport infrastructure were classified as “asphalt cells” and the rest were classified as “non-asphalt cells”. During each survey, the observer followed each raptor and recorded its position in the grid every five minutes. As the raptors were flying at low altitude (foraging flight, see above), their position was determined on aerial photographs and supported on easily recognizable landmarks, such as lonely trees or field borders that ensure enough accuracy in the 100 x 100 grid.

### Statistical analyses

#### Landscape scale

We first analyzed trends in raptor community as a whole by multidimensional scaling analysis (MDS). This technique creates new synthetic variables (dimensions) from the data on species presence and abundance, and projects the sampling plots on those new variables [44]. Sampling plots with similar community composition will appear nearby, while plots with different species or abundances will be apart. The distance matrix used to build the MDS was calculated using the Bray-Curtis index [44]. This index is commonly used in community analyses, as it uses information on abundance of each species to compute the distance matrix. We built a MDS with two dimensions to allow easier interpretation of results, after checking in the scree-plot of stress values that the inclusion of a third one did not improve stress noticeably (stress value for two dimensions: 0.25). Influence of each species was calculated by Spearman rank correlation of species abundance with both dimensions. Seasonal changes in raptor community and the effect of traffic, represented in a synthetic way by the types of infrastructure (control, road, motorway), were tested by a multivariate analysis of variance (MANOVA) and Tukey HSD post hoc tests. For these analyses, the locations of sampling plots in the two dimensions were used as response variables.

The relative relevance of transport infrastructure on foraging habitat selection of raptors was analyzed for the three community variables (diversity, abundance and richness), and for individual taxa by generalized linear mixed models (GLMMs) [45, 46], which were ranked and selected based on information-theoretic criteria [47, 48]. For each response variable, we created a set of candidate models based on ecological hypotheses. The hypotheses were divided into three groups: (i) General habitat structure, including main vegetation types, distance to nearest village and traffic volume of sampling plots; (ii) Food availability, including natural prey abundance as well as road casualty carcasses; (iii) Interaction with other raptor species, only for the species-level analyses, and including among the predictors the abundance of the most common species in the area: *M*. *milvus*, *M*. *migrans* and *H*. *pennatus*. The traffic predictor (ADT) was included in quadratic form, as vertebrates may show a non-linear response to traffic [49, 50]. The candidate set of models for each response variable included models representing one or more hypotheses through different combinations of predictors, using only those predictors relevant to that response (as described in the “variables used for landscape analysis” section).

Prior to model fit, we tested for correlation between predictor variables using Spearman’s rank correlation. No correlation higher than 0.7 was found, thus all variables were considered. We first explored the shape of the relationship between responses and predictors by generalized additive models (GAMs), using the full model and fitted smoothing splines with 3 degrees of freedom. Then, linearity was assessed by visual inspection of the partial residual plots [51] and when appropriate, logarithmic or quadratic transformations of predictor variables were applied. In order to avoid overparameterization, we included our controlling variables (*season*, *time of day*, *visibility*) only on those models for which they were informative.

GLMMs were fitted using gaussian error structure with identity link for the diversity models, and poisson error structure with log link for all the others. The identity of the sampling plot was used as random factor. When the response included a high number of zeros (*B*. *buteo*, *G*. *fulvus*, *A*. *monachus*), zero-inflated poisson (ZIP) distribution was used instead. ZIP distribution allows analyzing data that present more zeros than expected in a poisson distribution, avoiding potential overdispersion and bias in the parameter estimation due to the excessive number of zeros [46]. To model the ZIP distribution we followed the procedure used by Bolker et al. [52], including a single constant term across the entire model to account for zero-inflation across the data set [53, 54]. For poisson GLMMs, overdispersion was tested and found close to 1 in all cases, thus no correction was applied.

Candidate models were compared and ranked using Akaike Information Criterion corrected for small sample size (AICc). A null model containing only the intercept was also included in the candidate set for comparison. For further analyses and interpretation, we selected models within < 2 ΔAICc, i.e. the difference to the AICc of the best ranked model, as all of them should be considered competitive for interpretation [55]. Within the selected models, whenever two nested models differing only in one parameter were selected, they were considered redundant and we only included the one with the lowest AICc value to avoid overrepresentation of predictors [48, 55, 56].

Furthermore, we analyzed the relative importance of the explanatory variables for each response. The relative importance of a predictor was obtained by computing the summatory of Akaike weights (w_i_) of the models where the predictor was present, using only the set of selected models. Therefore, a predictor that appears in all the selected models will have the maximum value of 1, meaning that it is the most important predictor for the respective response variable within the group of analyzed predictors. To interpret the effect of the explanatory variables, for each response we calculated the average model and obtained the average coefficients with shrinkage [55]. As this method does not provide a reliable estimate for the standard deviation of coefficients [55], we decided to interpret coefficients with absolute values smaller than 0.01 as indicative of a lack of a relevant effect in biological terms.

### Infrastructure use analysis

Within the sampling plots, we evaluated the preference or avoidance of the infrastructure for each observed individual of a single species using a use versus availability approach [57–59]. The index of infrastructure use was built based on proportions, so we used GLMMs with binomial error distribution and logit link, including sampling plot identity as random factor. As we did in the previous analyses, we first checked for effects of controlling variables on the responses and included them when necessary. As our goal was to compare the response of the individuals to the infrastructure when they are in its proximity with the response of the species at the landscape scale, we only included traffic as predictor. We used the number of sightings per individual as a weighting variable to account for the variability in this measure (sightings ranging from one to six, see above). In order to test for lower or higher use of asphalt cells than their availability, the model intercept was forced to be the actual proportion of asphalt cells in each plot, representing the expected value if raptors used them randomly.

All statistical analyses were performed using R statistical software 3.0.3 [60]. GAM, GLMM and ZIGLMM models were fitted using mgcv [61], lme4 [62], and glmmADMB packages [54], respectively. AIC_c_ values and averaged coefficients were obtained with the MuMIn package [63]. All values presented in the results section refer to mean ± standard deviation, unless specified otherwise.

## Results

We recorded 743 raptors belonging to 18 different species (S1 Table). Nine species were observed in less than 15 occasions, and therefore they were included in the community-level analysis but discarded for the species-level analysis. One individual of rough-legged buzzard *Buteo rufinus* was also detected but removed from the dataset because it is considered a rare species in the area.

**Fig 2:**
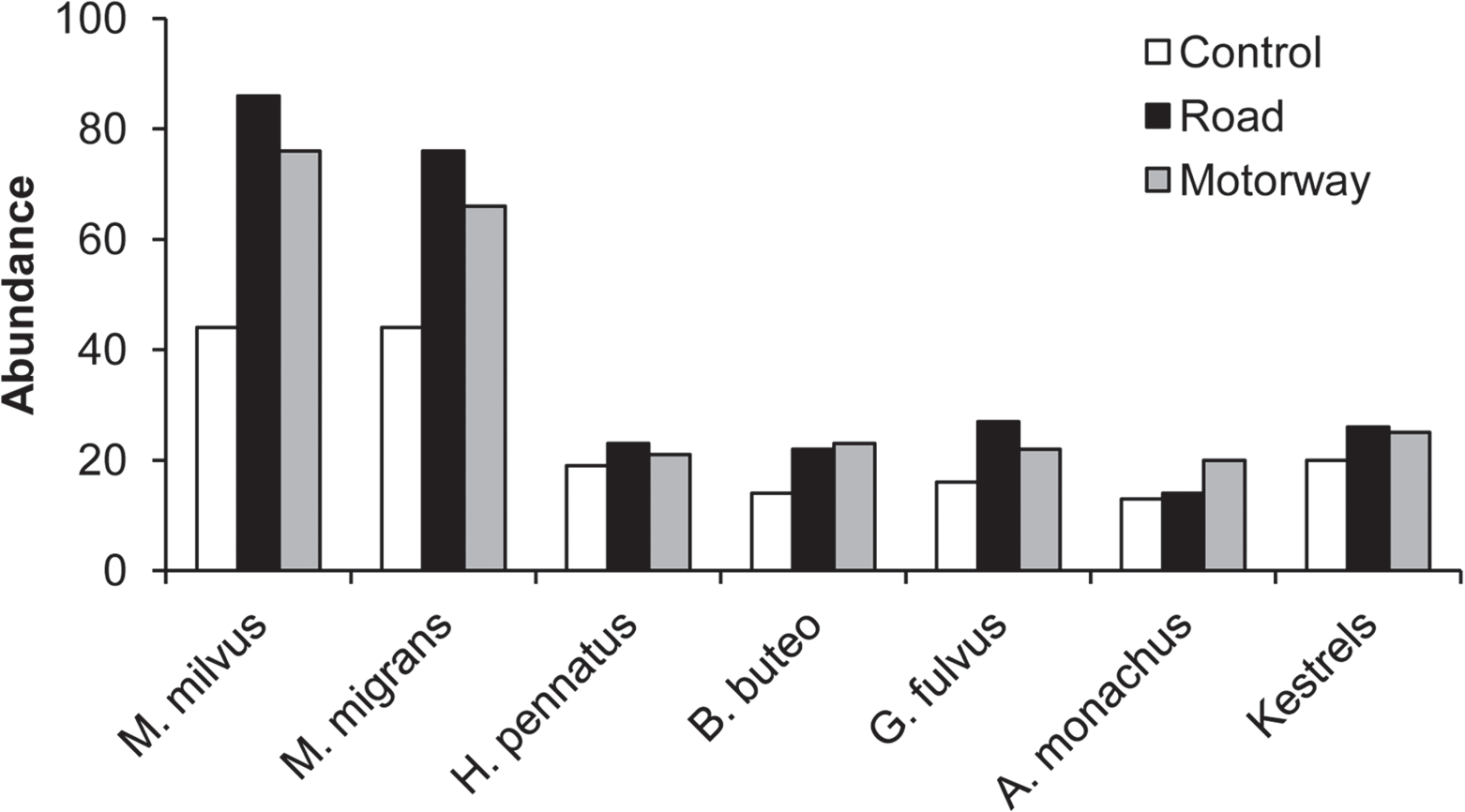
Species abundance. Total abundance of common raptor species in sampling plots of each type of transport infrastructure. Details for all the species detected in the study can be seen in S1 Table.

There was significant difference in abundance between types of plots (X = 9.24, df = 2, *p* = 0.009), but not for richness or diversity (X^2^ = 4.99, df = 2, *p* = 0.117; F = 1.59, df = 2, *p* = 0.213, respectively). We found a total of 187 raptors in control plots, 291 raptors in plots located near roads, and 265 raptors in motorways, with a similar significant abundance pattern in analyzed species (Fig. 2). The mean richness per plot was 1.63 ± 1.38 in control sites, 1.95 ± 1.35 in roads, and 2.05 ± 1.34 in motorways. In a parallel way, the diversity index was 0.46 ± 0.06 in control sites, 0.54 ± 0.06 in roads, and 0.60 ± 0.06 in motorways.

### Landscape scale analyses

**Fig 3:**
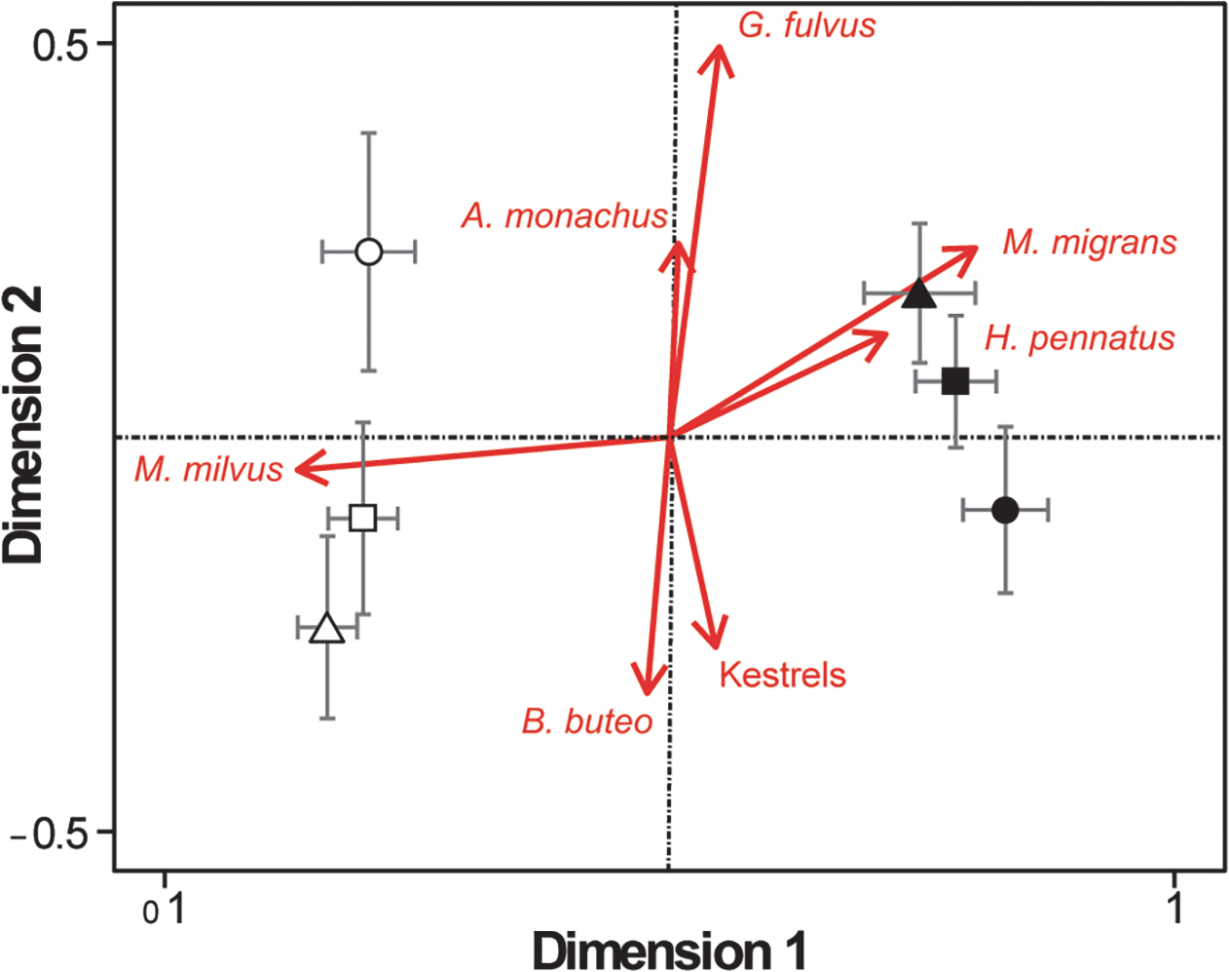
Two dimensional ordination (MDS) of general trends in raptor community. Species composition of sampling plots represented by transport infrastructure types and season (mean ± SE of position in dimensions 1 and 2). The influence of most abundant species is also represented with arrows of length and direction obtained from their correlation with the axes. Circles represent control plots, triangles road plots, and squares motorway plots. Solid symbols for breeding season and empty symbols for winter. Stress value of the ordination of 0.25.

The MDS analysis showed differences in raptor community composition linked to the presence of transport infrastructure and season (Fig. 3). There was a clear distinction in the community structure between breeding and winter seasons (F = 157.27, *p* < 0.001), and a significant interaction between season and transport infrastructure (F = 2.83, *p* = 0.025). In each season, we found the largest differences in community composition between road plots and control sites, being significant only in winter (*p* = 0.041), but not in all other cases (p > 0.05), and with motorway plots in an intermediate position (Fig. 3).

**Fig 4:**
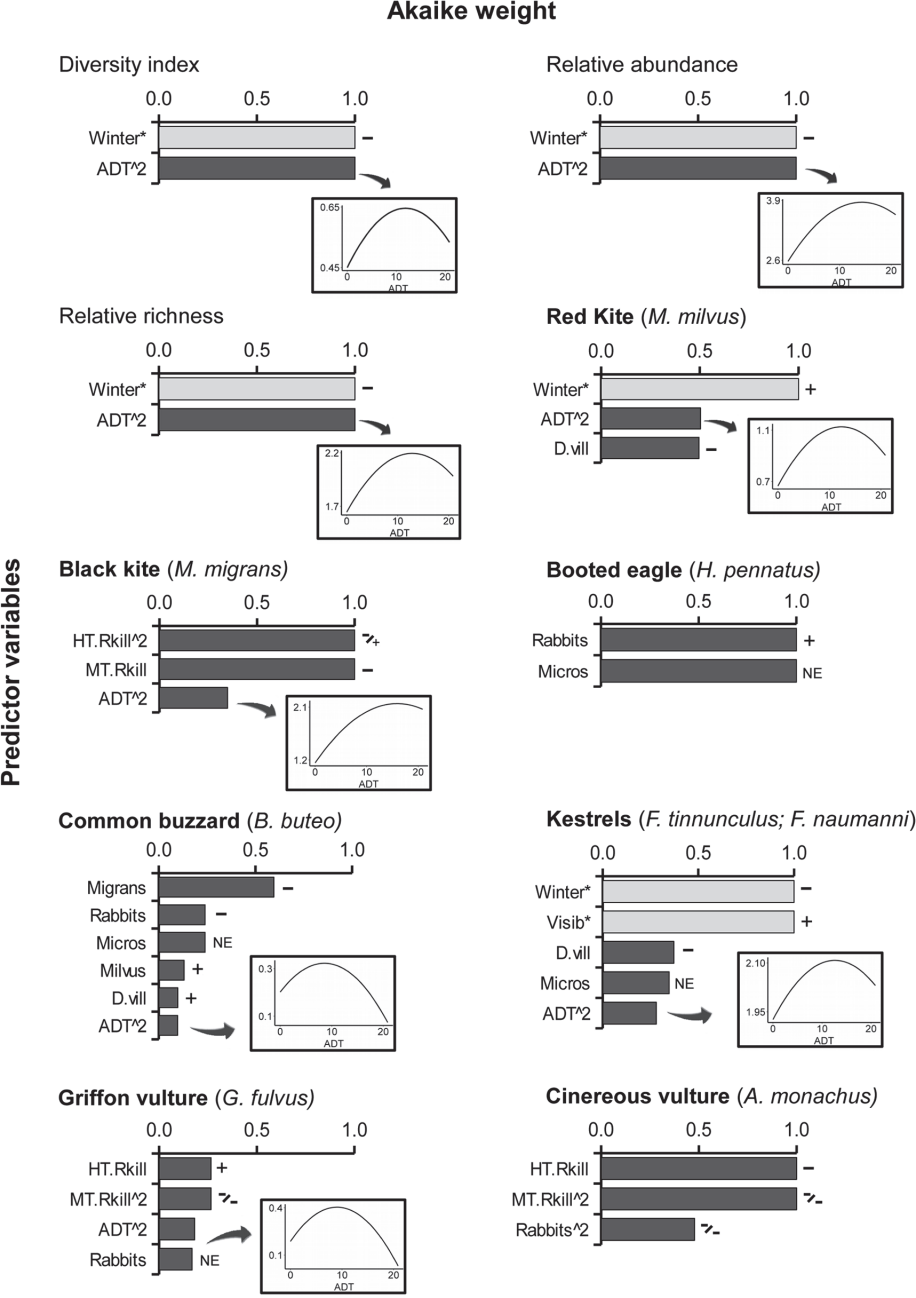
Relative importance of foraging habitat selection predictor variables at landscape scale. Akaike weights of predictors for each response variable in the community level analyses (diversity, abundance, richness) and the species-level analyses. Controlling variables are identified by asterisks and colored in light grey. The sign of the effect in the final average model is shown as positive (+) or negative (−). Variables in quadratic form are identified with “^2” and the symbols correspond to the simple and quadratic form, respectively. Variables with an averaged coefficient close to zero (≤|0.01|) are marked as “no effect” (NE). When the variable ADT is selected, the curve showing its effect in the response is also included. In this case, the x axis is always the amount of traffic (ADT), from 0 to 20.850 vehicles a day, and the y axis represents the value of the response variable. For a definition of the variables see Table 1 and the values of the averaged coefficients are in S10 and S11 Tables.

Regarding the habitat hypothesis at the community level, the most informative predictor variable for diversity, abundance and richness of raptors was the traffic volume (ADT) (Table 2. For the full list of models, see S2 Table). All three responses showed a quadratic response to traffic, reaching the maximum with medium traffic levels and then decreasing at highest traffic volume, especially in the case of diversity (Fig. 4, coefficients in S10 Table).

**Table 2:** Selected models for foraging habitat selection with traffic effects at the landscape scale.

Response	Hypothesis supported by model	AICc^1^	ΔAICc	w_i_ ^2^
**Community-level**				
Diversity	(i) Traffic volume	324.62	0.00	0.501
Abundance	(i) Traffic volume	1043.26	0.00	0.524
Richness	(i) Traffic volume	778.79	0.00	0.475
**Species-level**				
Red kite *(Milvus milvus)*	(i) Traffic volume	574.63	0.00	0.244
	(i)Distance to village	574.66	0.03	0.241
Black kite *(Milvus migrans)*	(ii) Anthropogenic food from roadkills	387.76	0.00	0.246
	(i) and (ii) Traffic volume and anthropogenic food from roadkills	389.00	1.24	0.132
Booted eagle *(Hieraaetus pennatus)*	(ii) Rabbit and micros abundance	238.17	0.00	0.430
Common buzzard *(Buteo buteo)*	(iii) Interaction with *M*. *migrans*	281.44	0.00	0.147
	(ii) and (iii) Rabbits and micros abundance and interaction with *M*. *migrans*	281.59	0.14	0.137
	(0) Intercept only model	282.21	0.77	0.100
	(iii) Interaction with *M*. *milvus*	282.80	1.36	0.075
	(i) Distance to village	283.38	1.94	0.056
	(i) and (iii) Traffic volume and interaction with *M*. *migrans*	283.39	1.95	0.056
Kestrels *(Falco tinnunculus*, *F*. *naumanii)*	(i) Distance to village	234.24	0.00	0.306
	(ii) Micros abundance	324.38	0.14	0.286
	(i) Traffic volume	324.81	0.57	0.231
Griffon vulture *(Gyps fulvus)*	(0) Intercept only model	303.97	0.00	0.267
	(ii) Anthropogenic food from roadkills	304.70	0.74	0.185
	(i) Traffic volume	305.47	1.50	0.126
	(ii) Rabbit abundance (carcasses)	305.61	1.64	0.117
Cinereous vulture *(Aegypius monachus)*	(ii) Anthropogenic food from roadkills	239.90	0.00	0.376
	(ii) Anthropogenic food from roadkills and Rabbit abundance (carcasses)	240.08	0.18	0.344

Models are presented based on the ecological hypothesis they support: (0) intercept only, (i) Habitat structure, (ii) Food availability, (iii) Interaction with other species. A full list of models can be found in supplementary material (S2–S11 Tables).

^1^AIC_c_ = Akaike Information Criterion value corrected for small sample size.

^2^w_i_ = Akaike weights of the models in the full set of candidate models.

In the species-specific analyses more than one model was selected for all taxa except booted eagles (Table 2). The variable that was selected for most species was the traffic volume –selected in 5 out of 7 species–, followed by the abundance of natural prey –selected in 4 species–, and the distance to anthropogenic areas, both villages and roadkill hotspots –selected in 3 species–. Also the null model was selected for common buzzard and griffon vulture, suggesting that the predictor variables had low explanatory power for them (for the full list of models for each species see S3–S9 Tables).

The Akaike weights of each explanatory variable for each response can be seen in Fig. 4 (see S11 Table for the whole set of averaged coefficient values). Response to traffic varied among species, both in relative importance and coefficient signs. Both kites and kestrels showed increasing abundance with low to medium traffic volumes (β_ADT, M.milvus_ = 1.301, β_ADT, M.migrans_ = 0.911, β_ADT, kestrels_ = 0.777). However, red kite and kestrels abundance decreased at higher traffic values (β_ADT^2^, M.milvus_ = −0.785, β_ADT^2^, kestrels_ = −0.619), and only black kite remained abundant (β_ADT^2^, M.migrans_ = 0.279). In the case of common buzzards and griffon vultures, although they had slightly higher abundance in areas with medium-low traffic than control sites (β_ADT, B.buteo_ = 0.101, β_ADT, G.fulvus_ = 0.215), their abundance decreased quickly with increasing traffic amounts (β_ADT^2^, B. buteo_ = −0.199, β_ADT^2^, G.fulvus_ = −0.762).

The roadkill hotspots were selected as important variables for species with the most marked scavenger behavior. Cinereous vultures were more abundant near hotspots in both medium and high traffic areas (β_L.HTRkill_ = −0.157, β_L.MTRkill_ = −9.300, β_L.MTRkill^2^_ = −6.971). Black kites and griffon vultures were abundant near roadkill hotspots located in medium traffic areas (β_L.MTRkill, M.migrans_ = −0.082, β_L.MTRkill, G.fulvus_ = −0.299, β_L.MTRkill^2^, G.fulvus_ = −2.098), but avoided those in high traffic areas areas (β_L.HTRkill, M.migrans_ = −0.034, β_L.HTRkill^2^, M.migrans_ = 3.379, β_L.HTRkill, G.fulvus_ = 0.012).

Red kites and kestrels selected areas close to villages, and common buzzards avoided them. Finally, in the case of booted eagle, no variable related to traffic was selected and its abundance was directly related to the abundance of rabbits (Fig. 4).

### Infrastructure use analysis

We analyzed the local behavior of individuals when observed inside infrastructure plots for 527 individuals belonging to the same taxa analyzed at the landscape scale. When the behavior was analyzed within 500 m of the infrastructure (radius of the sampling plot), red kites flew over the infrastructure more frequently than random for increasing traffic and griffon vultures avoided the infrastructure, especially with high traffic levels. For all other cases, we found no selection or avoidance of the infrastructure related to the amount of traffic (Table 3, for coefficients of the models see S12 Table).

**Table 3:** Raptors selection of asphalt cells within the sampling plots.

Species	ADT	ADT^2^
Red kite (*Milvus milvus*)	+	NS
Black kite (*Milvus migrans*)	NS	NS
Booted eagle (*Hieraaetus pennatus*)	NS	NS
Common buzzard (*Buteo buteo*)	NS	NS
Griffon vulture (*Gyps fulvus*)	-	-
Cinereous vulture (*Aegypius monachus*)	NS	NS
Kestrels (*Falco tinnunculus*, *F*. *naumanii*)	NS	NS

Models for red kites, common buzzard and kestrel included the controlling variable *season*, and the model for black kites included the controlling variable *time* (all coefficients in S12 Table.

+ = Preference, significant positive selection.

- = Avoidance, significant negative selection.

NS = Indifferent, no significant effect found.

## Discussion

We present the first comprehensive analysis of a diurnal raptor community aimed to compare the effects of transport infrastructure with other landscape factors in foraging habitat selection. The traffic volume of roads played a central role in the community metrics—diversity, abundance and richness—, and it was also the predictor selected most times in the species specific analyses, appearing in five out of seven species. As expected, individual species showed different degrees of tolerance toward traffic, from preference to avoidance, with the latter being especially strong for areas with high traffic volumes. In contrast, when comparing the response to traffic at different scales, we found no response to traffic for many species at the local scale. In our study, the effects of traffic were detectable at a broad scale, as predicted by the HHS hypothesis when analyzing the main habitat selection variables [30].

### Effects of transport infrastructure on foraging habitat selection of raptors at landscape scale

For the community metrics no other explanatory variable than traffic was included in the models, thus indicating that traffic volume was the main feature driving raptor community foraging habitat selection. Community metrics summarize responses from all the species and they reflect the most common one. However, they might fail to reflect ecological difference among species in disturbed landscapes [3], and finding common trends could thus be a difficult task for community metrics. Nonetheless, by calculating species-specific models we were able to disentangle effects on individual species and to find some common trends.

Five out of seven species (71.4%) showed a direct reaction to traffic, although the explanatory power of this predictor should be considered low for the two species that also included the null (intercept only) model (Table 2). Many species increased their abundance near areas with low to medium traffic densities, showing some tolerance to human disturbance and taking advantage of resources provided by roads, such as food or perching sites [24, 25]. The most human tolerant species, such as kites, showed the largest increase in abundance. These opportunistic species show high tolerance to human disturbances, being able to benefit from anthropogenic resources [24, 64], using their high maneuverability and fast reaction to avoid threats [65]. They can profit from food resources commonly found near roads, such as high prey density [8] or carcasses from roadkills [66]. The black kite was the only species that was not negatively affected by high traffic volumes, and also showed a strong selection for roadkill hotspots. Roads and motorways are areas easy to spot and for a generalist predator they could provide food in a more predictable way than random movements along the landscape, as it is suggested by the use of road verges described for some generalist predators, such as stone martens (*Martes foina*), red foxes (*Vulpes vulpes*) or raccoons (*Procyon lotor*) [67–69].

Contrary to other studies, we did not find a positive effect of roads per se on buzzards [24, 26]. The abundance of these species near roads could anyhow increase if the availability of perching sites and prey were higher in these places than at random [27, 33]. Also, the high frequency of zeros in our data could have influenced this result. However, it is unlikely that variables strictly related to roads, as the availability of carcasses, would attract these species because carrion is not a main part of their diet [23].

The griffon vulture also increases it abundance near low traffic areas, although rapidly declined when traffic increased, similar to results of Bautista et al [26]. Large species have greater alert distances and they need more time to initiate flight than smaller ones [65]. Therefore, a constant flow of incoming vehicles will make it difficult for them to use carcasses and thus they prefer areas with low or no traffic [28, 70]. Also, while our surveys were carried out there were no feeding stations for vultures in the study area and leaving carcasses from farms in the field is forbidden by European law [71]. Thus, finding food randomly distributed across the landscape might be a difficult task and, as it happens with other species, the proximity of roads may increase the likelihood of finding a carcass [72]. Vultures have been suffering from a shortage of food due to the removal of livestock carcasses by sanitary measures [71]. This situation together with non-optimal flight conditions for long distance flight like those of cold days in winter [72, 73], may have forced vultures to search for carrion in areas otherwise avoided, facing new risks that can deteriorate demographic parameters [74]. Under low food availability, vultures become more tolerant to taking risks [70] in order to exploit more predictable resources [75, 76], which would explain their attraction to roadkill hotspots. In addition, close to these areas there might be abundant carrion from injured animals that moved outside the road, or roadkilled ones that were projected some meters away after the impact with the vehicle. Besides, roadkill hotspots also reflect areas with high abundance of the roadkilled species, mainly rabbits, and proximity to these areas will increase the chance of finding dead or ill individuals to feed on.

In the case of the booted eagle, its abundance was driven by the abundance of prey, with no effect of roads or motorways. This species is tolerant to human presence [33], but it is an active predator that obtains food from hunting instead of scavenging [23]. If no avoidance of roads occurs, we can expect to find more eagles near roads only at those points with large prey populations nearby.

Although we only used the traffic volume in our models, this predictor was highly correlated with other traffic characteristics that might also explain raptors response to roads, such as speed limit. Sampling plots located in control areas had no traffic, road plots presented low to medium traffic with speed limit of 90 kmh^-1^, and motorway plots had medium to high traffic and speed limit of 120 kmh^-1^. As a general trend, the species were tolerant to traffic to some degree and when high traffic volume increased they showed a negative response, accordingly with the expectation of traffic density affecting vertebrate use of roads [26, 50], for which more tolerant species had higher thresholds. Raptors might perceive areas with more traffic as more dangerous places due to the higher speed limit [77], the disturbance created by noise [78], and the continuous flow of vehicles may prevent them from feeding on the surface.

Furthermore, the abundance patterns observed might be influenced not only by the average daily traffic, but as also by the specific traffic volume during the activity hours of raptors, the type of vehicles using the infrastructure, or the speed limit [77, 79]. Trucks could cause higher disturbance than smaller cars, especially if the peak traffic hours overlap with foraging activity of raptors. Further research is therefore needed to clarify the relationships of these variables in respect to raptor response.

### Response to transport infrastructure across scales

The patterns of selection or avoidance of the infrastructure at the landscape scale were not reflected in the analyses of local infrastructure use for many species. When focusing only in the area around infrastructure, we detected an effect of traffic for two species, the red kite, that used the infrastructure above its availability, and the griffon vulture, that avoided the infrastructure. These patterns are parallel to those of the broad scale, and they point to strong positive selection by kites and strong avoidance by vultures of roads when traffic increases. Kites probably feed close to the infrastructure, while vultures probably feed on carcasses that are not too close to the asphalt surface [28].

We did not detect any response for the other species, pointing to the possibility that the main habitat selection and therefore the response to roads happen at the broad scale [30]. Thus, the response of raptors to traffic may be difficult to assess when analyzing only individuals close to the infrastructure, as some individuals might use roads even if the species in general prefers areas with low traffic [13]. Also, individuals flying along the road also focus on the nearby terrain, and move away from the vertical of the asphalt surface for brief time periods to increase the amount of scanned surface for food (personal observation). Thus, punctual data on the location of birds might make difficult the detection of asphalt selection, even for individuals following the road. Besides, the lack of asphalt surface avoidance might be due to the low proportion that the roads occupy inside each observation plot (mean and SD of 18.5 ± 3.89%), thus the statistical detection of significant negative effects becomes difficult. Maybe a larger dataset or more specific methods to follow the raptor movements with accuracy over time (e.g. high resolution telemetry) would help to better understand raptors responses to roads at a local scale.

### Transport infrastructure and raptor conservation

As highlighted by our landscape level analyses, the effect of transport infrastructures was not restricted to the area above or adjacent to such infrastructures, but it extended into the landscape [12] with two main effects: Infrastructures reduced the habitat available to species avoiding traffic and attracted opportunistic and tolerant species. Also, some species may be attracted to roads by the presence of food, such as availability of prey or carrion, when there is few other sources in the landscape.

We should be cautious when interpreting the higher abundance of some species in certain areas affected by human activity. High abundances in risky areas could lead to negative effects on the population in the long term [17, 18, 80]. Collisions with cars are an important cause of mortality for birds [81], and raptors are more affected than other species [25]. Therefore, the availability of food can transform roads into population traps. In addition, by increasing the presence and abundance of generalist or human-tolerant species, human-modified landscapes promote biotic and functional homogenization [35, 82], at the cost of specialist species [34], and making species more dependent on resource subsidies [35]. Also, recent studies have shown that even for road selecting raptors, the stress caused by traffic can decrease their reproductive success [83].

Many raptor species show decreasing populations in recent years and are listed in the IUCN red list, including opportunistic species such as kites [20]. Measures to reduce food availability near roads while ensuring the presence of natural food resources in the landscape should be implemented to reduce avian exposure to traffic and mortality. Long term population effect studies are needed to assess the indirect effects of roads on them. Finally, raptors are top predators and they may control ecosystem dynamics by top-down regulation of prey, with potential changes in the whole community structure when the raptor community is modified [84].

## Supporting Information

S1 Table Frequency of individuals of the species observed. During each season, we surveyed 20 plots of each type (control, road, motorway), giving a total n of 80 per type, and a general total of 240 data, after the two winters and two breeding seasons.
(DOCX)

S2 Table Community analyses. Models explaining relative diversity, abundance and richness at landscape scale. Models are presented within one of the tested hypotheses: (0) intercept only, (i) Habitat structure, (ii) Food availability.
(DOCX)

S3 Table Species-specific analysis: red kite *(M*. *milvus)*. Landscape foraging habitat selection models for red kite. Models are presented within one of the tested hypotheses: (0) intercept only, (i) Habitat structure, (ii) Food availability, (iii) interaction with other species.
(DOCX)

S4 Table Species level analysis: *Milvus migrans*. Landscape foraging habitat selection models for black kite. Models are presented within one of the tested hypotheses: (0) intercept only, (i) Habitat structure, (ii) Food availability, (iii) Interaction with other species.
(DOCX)

S5 Table Species-specific analysis: booted eagle *(H*. *pennatus)*. Landscape foraging habitat selection models for booted eagle. Models are presented within one of the tested hypotheses: (0) intercept only, (i) Habitat structure, (ii) Food availability, (iii) interaction with other species.
(DOCX)

S6 Table Species-specific analysis: common buzzard *(B*. *buteo)*. Landscape foraging habitat selection models for common buzzard. Models are presented within one of the tested hypotheses: (0) intercept only, (i) Habitat structure, (ii) Food availability, (iii) interaction with other species.
(DOCX)

S7 Table Species-specific analysis: kestrels *(F*. *tinnunculus and F*. *naumanni)*. Landscape foraging habitat selection models for kestrels. Models are presented within one of the tested hypotheses: (0) intercept only, (i) Habitat structure, (ii) Food availability.
(DOCX)

S8 Table Species-specific analysis: griffon vulture *(G*. *fulvus)*. Landscape foraging habitat selection models for griffon vulture. Models are presented within one of the tested hypotheses: (0) intercept only, (i) Habitat structure, (ii) Food availability.
(DOCX)

S9 Table Species-specific analysis: cinereous vulture *(A*. *monachus)*. Landscape foraging habitat selection models for cinereous vulture. Models are presented within one of the tested hypotheses: (0) intercept only, (i) Habitat structure, (ii) Food availability.
(DOCX)

S10 Table Coefficients of community level models. Only variables in the selected models within 2 points of AICc were used.
(DOCX)

S11 Table Averaged coefficients of species-specific models. Only variables in the selected models within 2 points of AICc were used.
(DOCX)

S12 Table Model coefficients for raptor response to asphalt surface within the sampling plots. (DOCX)
