# Supplementary material for: Transport Infrastructure Shapes Foraging Habitat in a Raptor Community
Source: PLoS One. 2015 Mar 18;10(3):e0118604. doi: 10.1371/journal.pone.0118604 (PMC4365038; doi:10.1371/journal.pone.0118604)
Supplement: S1 Table — During each season, we surveyed 20 plots of each type (control, road, motorway), giving a total n of 80 per type, and a general total of 240 data, after the two winters and two breeding seasons. (DOCX) [file pone.0118604.s001.docx]

**S1 Table**. **Frequency of individuals of the species observed.** During each season, we surveyed 20 plots of each type (control, road, motorway), giving a total n of 80 per type, and a general total of 240 data, after the two winters and two breeding seasons.

| **Species** | | **Status^1^** | **CONTROL** | | | | |  | **ROAD** | | | | |  | **MOTORWAY** | | | | | **TOTAL** |
| --- | --- | --- | --- | --- | --- | --- | --- | --- | --- | --- | --- | --- | --- | --- | --- | --- | --- | --- | --- | --- |
|  |  |  | **B1** | **W1** | **B2** | **W2** | **Total** |  | **B1** | **W1** | **B2** | **W2** | **Total** |  | **B1** | **W1** | **B2** | **W2** | **Total** |  |
| Red Kite | *Milvus milvus* | Resident | 7 | 18 | 3 | 16 | 44 |  | 10 | 28 | 8 | 40 | 86 |  | 12 | 28 | 8 | 28 | 76 | 206 |
| Black Kite | *Milvus migrans* | Breeding | 24 | 0 | 20 | 0 | 44 |  | 40 | 0 | 36 | 0 | 76 |  | 37 | 0 | 29 | 0 | 66 | 186 |
| Common/ Lesser Kestrels | *Falco tinnunculus/ naumanni* | Resident/ Breeding | 6 | 3 | 8 | 3 | 20 |  | 14 | 4 | 5 | 3 | 26 |  | 6 | 6 | 5 | 8 | 25 | 71 |
| Griffon Vulture | *Gyps fulvus* | Resident | 2 | 6 | 1 | 7 | 16 |  | 3 | 9 | 7 | 3 | 22 |  | 7 | 14 | 4 | 2 | 27 | 65 |
| Booted Eagle | *Hieraaetus pennatus* | Breeding | 7 | 12 | 0 | 0 | 19 |  | 3 | 18 | 0 | 0 | 21 |  | 8 | 15 | 0 | 0 | 23 | 63 |
| Common Buzzard | *Buteo buteo* | Resident | 2 | 3 | 6 | 3 | 14 |  | 4 | 7 | 2 | 9 | 22 |  | 5 | 7 | 3 | 8 | 23 | 59 |
| Cinereous Vulture | *Aegypius monachus* | Resident | 1 | 2 | 7 | 3 | 13 |  | 2 | 3 | 3 | 6 | 14 |  | 6 | 6 | 6 | 2 | 20 | 47 |
| Montagu's Harrier | *Circus pygargus* | Breeding | 5 | 0 | 1 | 0 | 6 |  | 1 | 0 | 4 | 0 | 5 |  | 1 | 0 | 1 | 0 | 2 | 13 |
| Hen Harrier | *Circus cyaneus* | Resident | 0 | 1 | 0 | 0 | 1 |  | 0 | 3 | 0 | 1 | 4 |  | 0 | 2 | 0 | 0 | 2 | 7 |
| Golden Eagle | *Aquila chrysaetos* | Resident | 1 | 1 | 0 | 1 | 3 |  | 0 | 0 | 0 | 1 | 1 |  | 0 | 0 | 0 | 1 | 1 | 5 |
| Short-toed Eagle | *Circaetus gallicus* | Breeding | 0 | 0 | 1 | 0 | 1 |  | 0 | 0 | 0 | 0 | 0 |  | 2 | 0 | 2 | 0 | 4 | 5 |
| Spanish Imperial Eagle | *Aquila adalberti* | Resident | 0 | 1 | 0 | 0 | 1 |  | 0 | 2 | 1 | 0 | 3 |  | 0 | 0 | 0 | 0 | 0 | 4 |
| Western Marsh-harrier | *Circus aeruginosus* | Resident | 0 | 0 | 0 | 0 | 0 |  | 2 | 0 | 0 | 0 | 2 |  | 1 | 1 | 0 | 0 | 2 | 4 |
| Peregrine Falcon | *Falco peregrinus* | Resident | 1 | 0 | 2 | 0 | 3 |  | 0 | 0 | 1 | 0 | 1 |  | 0 | 0 | 0 | 0 | 0 | 4 |
| Eurasian Sparrowhawk | *Accipiter nisus* | Resident | 0 | 1 | 0 | 0 | 1 |  | 0 | 0 | 0 | 0 | 0 |  | 0 | 0 | 1 | 0 | 1 | 2 |
| Eurasian Hobby | *Falco subbuteo* | Breeding | 0 | 0 | 0 | 0 | 0 |  | 1 | 0 | 0 | 0 | 1 |  | 0 | 0 | 0 | 0 | 0 | 1 |
| Long-legged Buzzard* | *Buteo rufinus** | - | 0 | 1 | 0 | 0 | 1 |  | 0 | 0 | 0 | 0 | 0 |  | 0 | 0 | 0 | 0 | 0 | 1 |
| **Total** |  |  | 56 | 49 | 49 | 33 | 187 |  | 80 | 74 | 67 | 63 | 284 |  | 85 | 79 | 59 | 49 | 272 | 743 |

^1^ Status described in Sanz-Zuasti and Velasco (2001).

B = Breeding season; W = Winter season.

* Rare species.
