# Supplementary material for: Transport Infrastructure Shapes Foraging Habitat in a Raptor Community
Source: PLoS One. 2015 Mar 18;10(3):e0118604. doi: 10.1371/journal.pone.0118604 (PMC4365038; doi:10.1371/journal.pone.0118604)
Supplement: S2 Table — Models explaining relative diversity, abundance and richness at landscape scale. Models are presented within one of the tested hypotheses: (0) intercept only, (i) Habitat structure, (ii) Food availability. (DOCX) [file pone.0118604.s002.docx]

**S2 Table. Community analyses**. Models explaining relative diversity, abundance and richness at landscape scale. Models are presented within one of the tested hypotheses: (0) intercept only, (i) Habitat structure, (ii) Food availability.

|  |  | **Diversity** | | **Abundance** | | | **Richness** | | |
| --- | --- | --- | --- | --- | --- | --- | --- | --- | --- |
| **Predictors** | | **AICc** | **ΔAICc** | **Overdisp^1^** | **AICc** | **ΔAICc** | **Overdisp^1^** | **AICc** | **ΔAICc** |
| *(0) Null model* | |  |  |  |  |  |  |  |  |
|  | ~ 1 | 356.8 | 32.2 | 1.58 | 1083.8 | 40.6 | 1.20 | 807.8 | 29.0 |
| *(i) Habitat structure* | |  |  |  |  |  |  |  |  |
|  | ~ season + habitat + L. Dvill + adt^2 | 330.5 | 5.9 | 1.47 | 1052.8 | 9.5 | 1.07 | 786.2 | 7.4 |
|  | ~ season + adt^2 | 324.6 | 0.0 *S | 1.44 | 1043.3 | 0.0 *S | 1.06 | 778.8 | 0.0 *S |
|  | ~ season + habitat | 329.9 | 5.2 | 1.44 | 1052.3 | 9.1 | 1.08 | 784.0 | 5.3 |
|  | ~ season + L.Dvill | 326.8 | 2.2 | 1.42 | 1046.3 | 3.0 | 1.08 | 780.9 | 2.1 |
| *(ii) Food availability* | |  |  |  |  |  |  |  |  |
|  | ~ season +L. HTrkill + L.MTrkill + L.rabbits + micros | 333.2 | 8.6 | 1.44 | 1052.9 | 9.7 | 1.09 | 786.9 | 8.1 |
|  | ~ season + L.HTrkill + L.MTrkill | 329.2 | 4.5 | 1.43 | 1048.8 | 5.5 | 1.08 | 783.0 | 4.2 |
|  | ~ season + L. rabbits + micros | 329.8 | 5.2 | 1.43 | 1049.1 | 5.8 | 1.08 | 783.5 | 4.7 |
| *(i) and (ii) Habitat + Food* | |  |  |  |  |  |  |  |  |
|  | ~ season + habitat + L. Dvill + adt^2 + L.HTrkill + L.MTrkill + L. rabbits + micros | 338.3 | 13.7 | 1.50 | 1061.2 | 18.0 | 1.09 | 794.4 | 15.6 |
|  | ~ season + L.HTrkill + L.MTrkill + L. rabbits + micros + adt^2 | 332.6 | 7.9 | 1.46 | 1051.2 | 8.0 | 1.08 | 786.3 | 7.5 |
|  | ~ season + L.HTrkill + L.MTrkill + adt^2 | 328.9 | 4.3 | 1.45 | 1047.0 | 3.8 | 1.07 | 782.9 | 4.1 |
|  | ~ season + L. rabbits + micros * adt^2 | 330.8 | 6.2 | 1.47 | 1046.2 | 2.9 | 1.07 | 783.8 | 5.0 |
|  | ~ season + L. rabbits + micros + adt^2 | 328.2 | 3.6 | 1.45 | 1047.3 | 4.0 | 1.07 | 782.1 | 3.3 |

Diversity models follow gaussian distribution. Abundance and richness follow poisson distributions.

Variables marked with “^2” were included in the analyses in their quadratic form (variable + variable^2^).

All models include the identity of the observation point as random factor (1|Pt.ID).

* Models within Δ ≤ 2 of the best model. When nested models are included in this subset, only the model with lowest AICc is considered for further analyses.

S Models selected for averaging.

^1^ Overdispersion value.
