# Supplementary material for: Transport Infrastructure Shapes Foraging Habitat in a Raptor Community
Source: PLoS One. 2015 Mar 18;10(3):e0118604. doi: 10.1371/journal.pone.0118604 (PMC4365038; doi:10.1371/journal.pone.0118604)
Supplement: S3 Table — Landscape foraging habitat selection models for red kite. Models are presented within one of the tested hypotheses: (0) intercept only, (i) Habitat structure, (ii) Food availability, (iii) interaction with other species. (DOCX) [file pone.0118604.s003.docx]

**S3 Table. Species-specific analysis: red kite *(M. milvus)***. Landscape foraging habitat selection models for red kite. Models are presented within one of the tested hypotheses: (0) intercept only, (i) Habitat structure, (ii) Food availability, (iii) interaction with other species.

| **Predictors** | | **Overdisp^1^** | | **AICc** | | **ΔAICc** | |  | |  |
| --- | --- | --- | --- | --- | --- | --- | --- | --- | --- | --- |
| *(0) Null model* | | |  | |  | |  | |  | |
|  | ~ 1 | | 1.40 | | 637.2 | | 62.6 | |  | |
| *(i) Habitat structure* | | |  | |  | |  | |  | |
|  | ~ season + habitat + L.Dvill + adt^2 | | 1.22 | | 580.6 | | 5.9 | |  | |
|  | ~ season + adt^2 | | 1.17 | | 574.6 | | 0.0 | | *S | |
|  | ~ season + habitat | | 1.17 | | 584.1 | | 9.5 | |  | |
|  | ~ season + L.Dvill | | 1.16 | | 574.7 | | 0.1 | | *S | |
| *(ii) Food availability* | | |  | |  | |  | |  | |
|  | ~ season + L.HTrkill + L.MTrkill + L.rabbits + micros | | 1.18 | | 582.6 | | 7.9 | |  | |
|  | ~ season + L.HTrkill + L.MTrkill | | 1.15 | | 580.9 | | 6.3 | |  | |
|  | ~ season + L.rabbits + micros | | 1.17 | | 578.3 | | 3.7 | |  | |
| *(i) and (ii) Habitat + Food* | | |  | |  | |  | |  | |
|  | ~ season + habitat + L.Dvill + adt^2 + L.HTrkill + L.MTrkill + L.rabbits + micros | | 1.23 | | 587.5 | | 12.8 | |  | |
|  | ~ season + L.HTrkill + L.MTrkill + L.rabbits + micros + adt^2 | | 1.20 | | 580.6 | | 6.0 | |  | |
|  | ~ season + L.HTrkill + L.MTrkill + adt^2 | | 1.19 | | 578.0 | | 3.4 | |  | |
|  | ~ season + L.rabbits + micros * adt^2 | | 1.20 | | 580.6 | | 5.9 | |  | |
|  | ~ season + L.rabbits + micros + adt^2 | | 1.19 | | 577.3 | | 2.6 | |  | |
| *(iii) interaction with other species, habitat and food* | | |  | |  | |  | |  | |
|  | ~ season + habitat + adt^2 + L.Dvill + migrans | | 1.22 | | 582.6 | | 8.0 | |  | |
|  | ~ season + habitat + adt^2 + L.Dvill + pennatus | | 1.21 | | 582.5 | | 7.9 | |  | |
|  | ~ season + L.HTrkill + L.MTrkill + L.rabbits + micros + migrans | | 1.19 | | 584.5 | | 9.8 | |  | |
|  | ~ season + L.HTrkill + L.MTrkill + L.rabbits + micros + pennatus | | 1.17 | | 583.9 | | 9.3 | |  | |
|  | ~ season + L.HTrkill + L.MTrkill + migrans | | 1.16 | | 582.7 | | 8.1 | |  | |
|  | ~ season + L.HTrkill + L.MTrkill + pennatus | | 1.15 | | 582.5 | | 7.8 | |  | |
|  | ~ season + L.rabbits + micros + migrans | | 1.18 | | 580.2 | | 5.6 | |  | |
|  | ~ season + L.rabbits + micros + pennatus | | 1.16 | | 579.7 | | 5.0 | |  | |
|  | ~ season + adt^2 + migrans | | 1.18 | | 576.6 | | 1.9 | | * | |
|  | ~ season + adt^2 + pennatus | | 1.17 | | 576.3 | | 1.6 | | * | |
|  | ~ season + migrans | | 1.16 | | 579.1 | | 4.4 | |  | |
|  | ~ season + pennatus | | 1.14 | | 578.9 | | 4.3 | |  | |

All models follow poisson distribution and include the identity of the observation point as random factor (1|Pt.ID).

Variables marked with “^2” were included in the analyses in their quadratic form (variable + variable^2^).

* Models within Δ ≤ 2 of the best model. When nested models are included in this subset, only the model with lowest AICc is considered for further analyses.

S Models selected for averaging.

^1^ Overdispersion value.
