# Supplementary material for: Transport Infrastructure Shapes Foraging Habitat in a Raptor Community
Source: PLoS One. 2015 Mar 18;10(3):e0118604. doi: 10.1371/journal.pone.0118604 (PMC4365038; doi:10.1371/journal.pone.0118604)
Supplement: S4 Table — Landscape foraging habitat selection models for black kite. Models are presented within one of the tested hypotheses: (0) intercept only, (i) Habitat structure, (ii) Food availability, (iii) Interaction with other species. (DOCX) [file pone.0118604.s004.docx]

**S4 Table. Species level analysis: *Milvus migrans*.** Landscape foraging habitat selection models for black kite. Models are presented within one of the tested hypotheses: (0) intercept only, (i) Habitat structure, (ii) Food availability, (iii) Interaction with other species.

| **Predictors** | | **Overdisp^1^** | **AICc** | **ΔAICc** |  |  |  |
| --- | --- | --- | --- | --- | --- | --- | --- |
| *(0) Null model* | | |  |  |  |  | |
|  | | ~ 1 | 0.80 | 390.3 | 2.5 |  | |
| *(i) Habitat structure* | | |  |  |  |  | |
|  | | ~ habitat + L.Dvill + adt^2 | 0.88 | 399.1 | 11.4 |  | |
|  | | ~ adt^2 | 0.83 | 390.6 | 2.8 |  | |
|  | | ~ habitat | 0.84 | 397.1 | 9.4 |  | |
|  | | ~ L.Dvill | 0.81 | 390.1 | 2.3 |  | |
| *(ii) Food availability* | | |  |  |  |  | |
|  | | ~ L.HTrkill^2 + L.MTrkill + L.rabbits + micros | 0.90 | 390.7 | 2.9 |  | |
|  | | ~ L.HTrkill^2 + L.MTrkill | 0.87 | 387.8 | 0.0 | *S | |
|  | | ~ L.rabbits + micros | 0.83 | 392.9 | 5.1 |  | |
| *(i) and (ii) Habitat + Food* | | |  |  |  |  | |
|  | | ~ habitat + L.Dvill + adt^2+ L.HTrkill^2 + L.MTrkill + L.rabbits + micros | 0.98 | 402.3 | 14.5 |  | |
|  | | ~ L.HTrkill^2 + L.MTrkill + L.rabbits + micros + adt^2 | 0.91 | 393.2 | 5.4 |  | |
|  | | ~ L.HTrkill^2 + L.MTrkill + adt^2 | 0.89 | 389.0 | 1.2 | *S | |
|  | | ~ L.rabbits + micros * adt^2 | 0.88 | 399.0 | 11.3 |  | |
|  | | ~ L.rabbits + micros + adt^2 | 0.85 | 394.7 | 7.0 |  | |
| *(iii) interaction with other species, habitat and food* | | |  |  |  |  | |
|  | | ~ habitat + adt^2 + L.Dvill + milvus | 0.88 | 401.5 | 13.7 |  | |
|  | | ~ habitat + adt^2 + L.Dvill + pennatus | 0.89 | 401.5 | 13.7 |  | |
|  | | ~ L.HTrkill^2 + L.MTrkill + L.rabbits + micros + milvus | 0.91 | 392.9 | 5.1 |  | |
|  | | ~ L.HTrkill^2 + L.MTrkill + L.rabbits + micros + pennatus | 0.91 | 393.0 | 5.2 |  | |
|  | | ~ L.HTrkill^2 + L.MTrkill + milvus | 0.88 | 389.9 | 2.2 |  | |
|  | | ~ L.HTrkill^2 + L.MTrkill + pennatus | 0.89 | 389.9 | 2.1 |  | |
|  | | ~ L.rabbits + micros + milvus | 0.84 | 394.8 | 7.0 |  | |
|  | | ~ L.rabbits + micros + pennatus | 0.83 | 395.0 | 7.3 |  | |
|  | | ~ adt^2 + milvus | 0.84 | 392.7 | 5.0 |  | |
|  | | ~ adt^2 + pennatus | 0.84 | 392.8 | 5.0 |  | |
|  | | ~ milvus | 0.81 | 392.1 | 4.3 |  | |
|  | | ~ pennatus | 0.81 | 392.4 | 4.6 |  | |

All models follow poisson distribution and include the identity of the observation point as random factor (1|Pt.ID).

Variables marked with “^2” were included in the analyses in their quadratic form (variable + variable^2^).

* Models within Δ ≤ 2 of the best model. When nested models are included in this subset, only the model with lowest AICc is considered for further analyses.

S Models selected for averaging.

^1^ Overdispersion value.
