# Supplementary material for: Transport Infrastructure Shapes Foraging Habitat in a Raptor Community
Source: PLoS One. 2015 Mar 18;10(3):e0118604. doi: 10.1371/journal.pone.0118604 (PMC4365038; doi:10.1371/journal.pone.0118604)
Supplement: S5 Table — Landscape foraging habitat selection models for booted eagle. Models are presented within one of the tested hypotheses: (0) intercept only, (i) Habitat structure, (ii) Food availability, (iii) interaction with other species. (DOCX) [file pone.0118604.s005.docx]

**S5 Table. Species-specific analysis: booted eagle *(H. pennatus)***. Landscape foraging habitat selection models for booted eagle. Models are presented within one of the tested hypotheses: (0) intercept only, (i) Habitat structure, (ii) Food availability, (iii) interaction with other species.

| **Predictors** | | **Overdisp^1^** | **AICc** | **ΔAICc** |  |  |  |
| --- | --- | --- | --- | --- | --- | --- | --- |
| *(0) Null model* | | |  |  |  |  | |
|  | | ~ 1 | 0.98 | 243.2 | 5.1 |  | |
| *(i) Habitat structure* | | |  |  |  |  | |
|  | | ~ habitat + L.Dvill^2 + adt^2 | 1.11 | 253.9 | 15.7 |  | |
|  | | ~ adt^2 | 1.02 | 245.2 | 7.1 |  | |
|  | | ~ habitat | 1.03 | 249.7 | 11.5 |  | |
|  | | ~ L.Dvill^2 | 1.00 | 244.5 | 6.3 |  | |
| *(ii) Food availability* | | |  |  |  |  | |
|  | | ~ L.rabbits + micros | 1.05 | 238.2 | 0.0 | *S | |
| *(i) and (ii) Habitat + Food* | | |  |  |  |  | |
|  | | ~ habitat + L.Dvill^2 + adt^2 + L.rabbits + micros | 1.17 | 249.1 | 10.9 |  | |
|  | | ~ L.rabbits + micros * adt^2 | 1.11 | 242.1 | 3.9 |  | |
|  | | ~ L.rabbits + micros + adt^2 | 1.07 | 241.8 | 3.7 |  | |
| *(iii) interaction with other species, habitat and food* | | |  |  |  |  | |
|  | | ~ habitat + adt^2 + L.Dvill^2 + milvus | 1.12 | 256.0 | 17.8 |  | |
|  | | ~ habitat + adt^2 + L.Dvill^2 + migrans | 1.18 | 254.9 | 16.8 |  | |
|  | | ~ L.rabbits + micros + milvus | 1.07 | 240.1 | 1.9 | * | |
|  | | ~ L.rabbits + micros + migrans | 1.08 | 240.1 | 1.9 | * | |
|  | | ~ adt^2 + milvus | 1.03 | 247.2 | 9.1 |  | |
|  | | ~ adt^2 + migrans | 1.06 | 246.9 | 8.7 |  | |
|  | | ~ milvus | 0.99 | 245.0 | 6.9 |  | |
|  | | ~ migrans | 1.01 | 244.7 | 6.5 |  | |

All models follow poisson distribution and include the identity of the observation point as random factor (1|Pt.ID).

Variables marked with “^2” were included in the analyses in their quadratic form (variable + variable^2^).

* Models within Δ ≤ 2 of the best model. When nested models are included in this subset, only the model with lowest AICc is considered for further analyses.

S Models selected for averaging.

^1^ Overdispersion value.
