# Supplementary material for: Transport Infrastructure Shapes Foraging Habitat in a Raptor Community
Source: PLoS One. 2015 Mar 18;10(3):e0118604. doi: 10.1371/journal.pone.0118604 (PMC4365038; doi:10.1371/journal.pone.0118604)
Supplement: S6 Table — Landscape foraging habitat selection models for common buzzard. Models are presented within one of the tested hypotheses: (0) intercept only, (i) Habitat structure, (ii) Food availability, (iii) interaction with other species. (DOCX) [file pone.0118604.s006.docx]

**S6 Table. Species-specific analysis: common buzzard *(B. buteo)***. Landscape foraging habitat selection models for common buzzard. Models are presented within one of the tested hypotheses: (0) intercept only, (i) Habitat structure, (ii) Food availability, (iii) interaction with other species.

| **Predictors** | | **Overdisp^1^** | **AICc** | **ΔAICc** |  |  |  |
| --- | --- | --- | --- | --- | --- | --- | --- |
| *(0) Null model* | | |  |  |  |  | |
|  | | ~ 1 | 0.77 | 282.2 | 0.8 | *S | |
| *(i) Habitat structure* | | |  |  |  |  | |
|  | | ~ habitat + L.Dvill + adt^2 | 0.77 | 289.5 | 8.0 |  | |
|  | | ~ adt^2 | 0.76 | 284.7 | 3.2 |  | |
|  | | ~ habitat | 0.77 | 284.7 | 3.3 |  | |
|  | | ~ L.Dvill | 0.77 | 283.4 | 1.9 | *S | |
| *(ii) Food availability* | | |  |  |  |  | |
|  | | ~ L.HTrkill + L.MTrkill + L.rabbits + micros | 0.78 | 286.7 | 5.3 |  | |
|  | | ~ L.HTrkill + L.MTrkill | 0.77 | 286.1 | 4.7 |  | |
|  | | ~ L.rabbits + micros | 0.77 | 282.5 | 1.0 | * | |
| *(i) and (ii) Habitat + Food* | | |  |  |  |  | |
|  | | ~ habitat + L.Dvill + adt^2 + L.HTrkill + L.MTrkill + L.rabbits + micros | 0.78 | 295.1 | 13.7 |  | |
|  | | ~ L.HTrkill + L.MTrkill + L.rabbits + micros + adt^2 | 0.77 | 290.6 | 9.1 |  | |
|  | | ~ L.HTrkill + L.MTrkill + adt^2 | 0.76 | 288.7 | 7.3 |  | |
|  | | ~ L.rabbits + micros * adt^2 | 0.78 | 290.7 | 9.3 |  | |
|  | | ~ L.rabbits + micros + adt^2 | 0.77 | 286.5 | 5.1 |  | |
| *(iii) interaction with other species, habitat and food* | | |  |  |  |  | |
|  | | ~ habitat + adt^2 + L.Dvill + milvus | 0.79 | 290.7 | 9.3 |  | |
|  | | ~ habitat + adt^2 + L.Dvill + migrans | 0.78 | 289.2 | 7.8 |  | |
|  | | ~ habitat + adt^2 + L.Dvill + pennatus | 0.77 | 291.7 | 10.2 |  | |
|  | | ~ L.HTrkill + L.MTrkill + L.rabbits + micros + milvus | 0.77 | 287.3 | 5.8 |  | |
|  | | ~ L.HTrkill + L.MTrkill + L.rabbits + micros + migrans | 0.77 | 285.8 | 4.3 |  | |
|  | | ~ L.HTrkill + L.MTrkill + L.rabbits + micros + pennatus | 0.77 | 288.8 | 7.3 |  | |
|  | | ~ L.HTrkill + L.MTrkill + milvus | 0.77 | 286.8 | 5.3 |  | |
|  | | ~ L.HTrkill + L.MTrkill + migrans | 0.77 | 285.5 | 4.1 |  | |
|  | | ~ L.HTrkill + L.MTrkill + pennatus | 0.77 | 288.1 | 6.6 |  | |
|  | | ~ L.rabbits + micros + milvus | 0.76 | 283.0 | 1.6 | * | |
|  | | ~ L.rabbits + micros + migrans | 0.75 | 281.6 | 0.1 | *S | |
|  | | ~ L.rabbits + micros + pennatus | 0.76 | 284.5 | 3.0 |  | |
|  | | ~ adt^2 + milvus | 0.76 | 285.5 | 4.0 |  | |
|  | | ~ adt^2 + migrans | 0.75 | 283.4 | 1.9 | *S | |
|  | | ~ adt^2 + pennatus | 0.76 | 286.7 | 5.3 |  | |
|  | | ~ milvus | 0.77 | 282.8 | 1.4 | *S | |
|  | | ~ migrans | 0.77 | 281.4 | 0.0 | *S | |
|  | | ~ pennatus | 0.77 | 284.1 | 2.7 |  | |

All models follow zero-inflated poisson distribution and include the identity of the observation point as random factor (1|Pt.ID).

Variables marked with “^2” were included in the analyses in their quadratic form (variable + variable^2^).

* Models within Δ ≤ 2 of the best model. When nested models are included in this subset, only the model with lowest AICc is considered for further analyses.

S Models selected for averaging.

^1^ Overdispersion value.
