# Supplementary material for: Transport Infrastructure Shapes Foraging Habitat in a Raptor Community
Source: PLoS One. 2015 Mar 18;10(3):e0118604. doi: 10.1371/journal.pone.0118604 (PMC4365038; doi:10.1371/journal.pone.0118604)
Supplement: S7 Table — Landscape foraging habitat selection models for kestrels. Models are presented within one of the tested hypotheses: (0) intercept only, (i) Habitat structure, (ii) Food availability. (DOCX) [file pone.0118604.s007.docx]

**S7 Table. Species-specific analysis: kestrels *(F. tinnunculus and F. naumanni)***. Landscape foraging habitat selection models for kestrels. Models are presented within one of the tested hypotheses: (0) intercept only, (i) Habitat structure, (ii) Food availability.

| **Predictors** | | **Overdisp^1^** | **AICc** | **ΔAICc** |  |  |  |
| --- | --- | --- | --- | --- | --- | --- | --- |
| *(0) Null model* | | |  |  |  |  | |
|  | | ~ 1 | 0.65 | 334.3 | 10.1 |  | |
| *(i) Habitat structure* | | |  |  |  |  | |
|  | | ~ season + p.visib + habitat + L.Dvill + adt^2 | 0.65 | 332.4 | 8.2 |  | |
|  | | ~ season + p.visib + adt^2 | 0.65 | 324.8 | 0.6 | *S | |
|  | | ~ season + p.visib + habitat | 0.66 | 328.3 | 4.0 |  | |
|  | | ~ season + p.visib + L.Dvill | 0.66 | 324.2 | 0.0 | *S | |
| *(ii) Food availability* | | |  |  |  |  | |
|  | | ~ season + p.visib + micros | 0.66 | 324.4 | 0.1 | *S | |
| *(i) and (ii) Habitat + Food* | | |  |  |  |  | |
|  | | ~ season + p.visib + habitat + L.Dvill + adt^2 + micros | 0.65 | 334.6 | 10.4 |  | |
|  | | ~ season + p.visib + micros * adt^2 | 0.65 | 328.0 | 3.8 |  | |
|  | | ~ season + p.visib + micros + adt^2 | 0.65 | 326.9 | 2.7 |  | |

All models follow poisson distribution and include the identity of the observation point as random factor (1|Pt.ID).

Variables marked with “^2” were included in the analyses in their quadratic form (variable + variable^2^).

* Models within Δ ≤ 2 of the best model. When nested models are included in this subset, only the model with lowest AICc is considered for further analyses.

S Models selected for averaging.

^1^ Overdispersion value.
