# Supplementary material for: Transport Infrastructure Shapes Foraging Habitat in a Raptor Community
Source: PLoS One. 2015 Mar 18;10(3):e0118604. doi: 10.1371/journal.pone.0118604 (PMC4365038; doi:10.1371/journal.pone.0118604)
Supplement: S8 Table — Landscape foraging habitat selection models for griffon vulture. Models are presented within one of the tested hypotheses: (0) intercept only, (i) Habitat structure, (ii) Food availability. (DOCX) [file pone.0118604.s008.docx]

**S8 Table. Species-specific analysis: griffon vulture *(G. fulvus)***. Landscape foraging habitat selection models for griffon vulture. Models are presented within one of the tested hypotheses: (0) intercept only, (i) Habitat structure, (ii) Food availability.

| **Predictors** | | **Overdisp^1^** | **AICc** | **ΔAICc** |  |  |  |
| --- | --- | --- | --- | --- | --- | --- | --- |
| *(0) Null model* | | |  |  |  |  | |
|  | | ~ 1 | 0.981 | 303.966 | 0.000 | *S | |
| *(i) Habitat structure* | | |  |  |  |  | |
|  | | ~ habitat + adt^2 | 0.839 | 312.211 | 8.245 |  | |
|  | | ~ adt^2 | 0.842 | 305.470 | 1.505 | *S | |
|  | | ~ habitat | 0.896 | 311.149 | 7.183 |  | |
| *(ii) Food availability* | | |  |  |  |  | |
|  | | ~ L.HTrkill + poly(L.MTrkill,2) + L.rabbits | 0.900 | 306.689 | 2.723 |  | |
|  | | ~ L.HTrkill + poly(L.MTrkill,2) | 0.951 | 304.703 | 0.737 | *S | |
|  | | ~ L.rabbits | 0.902 | 305.610 | 1.645 | *S | |
| *(i) and (ii) Habitat + Food* | | |  |  |  |  | |
|  | | ~ habitat + adt^2 + L.HTrkill + poly(L.MTrkill,2) + L.rabbits | 0.746 | 316.461 | 12.495 |  | |
|  | | ~ L.HTrkill + poly(L.MTrkill,2) + L.rabbits + adt^2 | 0.760 | 308.223 | 4.257 |  | |
|  | | ~ L.HTrkill + poly(L.MTrkill,2) + adt^2 | 0.847 | 306.783 | 2.818 |  | |
|  | | ~ L.rabbits * adt^2 | 1.054 | 306.801 | 2.836 |  | |
|  | | ~ L.rabbits + adt^2 | 0.747 | 306.879 | 2.913 |  | |

All models follow zero-inflated poisson distribution and include the identity of the observation point as random factor (1|Pt.ID).

Variables marked with “^2” were included in the analyses in their quadratic form (variable + variable^2^).

* Models within Δ ≤ 2 of the best model. When nested models are included in this subset, only the model with lowest AICc is considered for further analyses.

S Models selected for averaging.

^1^ Overdispersion value.
