# Supplementary material for: Transport Infrastructure Shapes Foraging Habitat in a Raptor Community
Source: PLoS One. 2015 Mar 18;10(3):e0118604. doi: 10.1371/journal.pone.0118604 (PMC4365038; doi:10.1371/journal.pone.0118604)
Supplement: S9 Table — Landscape foraging habitat selection models for cinereous vulture. Models are presented within one of the tested hypotheses: (0) intercept only, (i) Habitat structure, (ii) Food availability. (DOCX) [file pone.0118604.s009.docx]

**S9 Table. Species-specific analysis: cinereous vulture *(A. monachus)***. Landscape foraging habitat selection models for cinereous vulture. Models are presented within one of the tested hypotheses: (0) intercept only, (i) Habitat structure, (ii) Food availability.

| **Predictors** | | **Overdisp^1^** | **AICc** | **ΔAICc** |  |  |  |
| --- | --- | --- | --- | --- | --- | --- | --- |
| *(0) Null model* | | |  |  |  |  | |
|  | | ~ 1 | 0.947 | 243.798 | 3.899 |  | |
| *(i) Habitat structure* | | |  |  |  |  | |
|  | | ~ habitat + adt^2 | 0.780 | 249.287 | 9.388 |  | |
|  | | ~ adt^2 | 0.900 | 245.718 | 5.820 |  | |
|  | | ~ habitat | 0.789 | 245.573 | 5.674 |  | |
| *(ii) Food availability* | | |  |  |  |  | |
|  | | ~ L.HTrkill + L.MTrkill^2 + L.rabbits^2 | 0.731 | 240.077 | 0.179 | *S | |
|  | | ~ L.HTrkill + L.MTrkill^2 | 0.800 | 239.899 | 0.000 | *S | |
|  | | ~ L.rabbits^2 | 0.898 | 245.048 | 5.150 |  | |
| *(i) and (ii) Habitat + Food* | | |  |  |  |  | |
|  | | ~ habitat + adt^2 + L.HTrkill + L.MTrkill^2 + L.rabbits^2 | 0.698 | 248.593 | 8.694 |  | |
|  | | ~ L.HTrkill + L.MTrkill^2 + L.rabbits^2 + adt^2 | 0.739 | 243.909 | 4.010 |  | |
|  | | ~ L.HTrkill + L.MTrkill^2 + adt^2 | 0.803 | 242.915 | 3.017 |  | |
|  | | ~ L.rabbits^2 * adt^2 | 0.823 | 254.450 | 14.551 |  | |
|  | | ~ L.rabbits^2 + adt^2 | 0.836 | 246.517 | 6.618 |  | |

All models follow zero-inflated poisson distribution and include the identity of the observation point as random factor (1|Pt.ID).

Variables marked with “^2” were included in the analyses in their quadratic form (variable + variable^2^).

* Models within Δ ≤ 2 of the best model. When nested models are included in this subset, only the model with lowest AICc is considered for further analyses.

S Models selected for averaging.

^1^ Overdispersion value.
