# Supplementary material for: Transport Infrastructure Shapes Foraging Habitat in a Raptor Community
Source: PLoS One. 2015 Mar 18;10(3):e0118604. doi: 10.1371/journal.pone.0118604 (PMC4365038; doi:10.1371/journal.pone.0118604)
Supplement: S10 Table — Only variables in the selected models within 2 points of AICc were used. (DOCX) [file pone.0118604.s010.docx]

**S10 Table**. C**oefficients of community level models**. Only variables in the selected models within 2 points of AICc were used.

|  | **Variable** | **Diversity** | **Abundance** | | **Richness** | |
| --- | --- | --- | --- | --- | --- | --- |
|  | (Intercept) | 0.713 | 1.263 | | 0.853 | |
| *Controlling variables* | |  |  |  | |  |
|  | Season [Winter] | -0.361 | -0.473 | | -0.528 | |
| *(i) Presence of roads or motorways* | | |  |  | |  |
|  | ADT | 0.714 | 1.998 | | 1.216 | |
|  | ADT^2^ | -0.841 | -1.260 | | -1.090 | |

Explanatory variables are grouped by the hypothesis they belong: (i) Habitat structure. Values for the intercept and controlling variables are also included although they are not considered informative parameters for these analyses. For a definition of the variables see table 1.
