# Supplementary material for: Transport Infrastructure Shapes Foraging Habitat in a Raptor Community
Source: PLoS One. 2015 Mar 18;10(3):e0118604. doi: 10.1371/journal.pone.0118604 (PMC4365038; doi:10.1371/journal.pone.0118604)
Supplement: S11 Table — Only variables in the selected models within 2 points of AICc were used. (DOCX) [file pone.0118604.s011.docx]

**S11 Table**. **Averaged coefficients of species-specific models**. Only variables in the selected models within 2 points of AICc were used.

|  | **Variable** | **Red kite**  ***M. milvus*** | | **Black kite**  ***M. migrans*** | **Booted eagle**  ***H. pennatus*** | | | | **Common buzzard**  ***B. buteo*** | | **Kestrels** | | **Griffon vulture**  ***G. fulvus*** | | **Cinereous vulture**  ***A. monachus*** | | |  |
| --- | --- | --- | --- | --- | --- | --- | --- | --- | --- | --- | --- | --- | --- | --- | --- | --- | --- | --- |
|  | (Intercept) | -0.170 | | 1.013 | -1.277 | | | | -0.486 | | -8.142 | | -0.183 | | 0.601 | | |  |
| *Controlling variables* | | |  | | |  | |  | | | |  |  | | | |  | |
|  | Season [Winter] | 1.193 | |  |  | | | |  | | -0.489 | |  | |  | | |  |
|  | Visib |  | |  |  | | | |  | | 7.569 | |  | |  | | |  |
| *(i) Habitat struture* | | | | | | |  | | |  | | | |  | |  | |  |
|  | ADT | 1.301 | | 0.911 |  | | | | 0.101 | | 0.777 | | 0.215 | |  | | |  |
|  | ADT^2^ | -0.785 | | 0.279 |  | | | | -0.199 | | -0.619 | | -0.762 | |  | | |  |
|  | L.Dvill | -0.108 | |  |  | | | | 0.021 | | -0.041 | |  | |  | | |  |
| *(ii) Food availability: Natural prey and anthropogenic food resources (roadkill)* | | | | | | | | | | | | | | | |  | |  |
|  | Rabbits |  | |  | 0.212 | | | | -0.021 | |  | | -0.010 | | -3.450 | | |  |
|  | Rabbits^2^ |  | |  |  | | | |  | |  | |  | | -5.132 | | |  |
|  | Voles |  | |  | NE | | | | NE | | NE | |  | |  | | |  |
|  | HT.Rkill |  | | -0.034 |  | | | |  | |  | | 0.012 | | -0.157 | | |  |
|  | HT.Rkill^2^ |  | | 3.379 |  | | | |  | |  | |  | |  | | |  |
|  | MT. Rkill |  | | -0.082 |  | | | |  | |  | | -0.299 | | -9.300 | | |  |
|  | MT. Rkill^2^ |  | |  |  | | | |  | |  | | -2.098 | | -6.971 | | |  |
| *(iii) Interaction with other species* | | | | | | |  | | |  | | | |  | |  | |  |
|  | Milvus |  | |  |  | | | | 0.018 | |  | |  | |  | | |  |
|  | Migrans |  | |  |  | | | | -0.128 | |  | |  | | 0.094 | | |  |

Explanatory variables are grouped by the hypothesis they belong: (i) Habitat structure, (ii) Food availability, (iii) Interaction with other species. Interactions between variables only occur between the variable *zone* and other variables and are included in the group of the latter. Values for the intercept and controlling variables are also included although they are not considered informative parameters for these analyses. Variables with an averaged coefficient close to zero (≤|0.01|) are marked as NE (no effect). For a definition of the variables see table S1.
