# Supplementary material for: Transport Infrastructure Shapes Foraging Habitat in a Raptor Community
Source: PLoS One. 2015 Mar 18;10(3):e0118604. doi: 10.1371/journal.pone.0118604 (PMC4365038; doi:10.1371/journal.pone.0118604)
Supplement: S12 Table — (DOCX) [file pone.0118604.s012.docx]

**S12 Table**. **Model coefficients for raptor response to asphalt surface within the sampling plots.**

|  | **adt** | **adt^2** | **Season**  **[Summer]^c^** | **Season**  **[Winter]^c^** | **Time**  **[Morning]^c^** | **Time**  **[Afternoon]^c^** |
| --- | --- | --- | --- | --- | --- | --- |
| *M .milvus* | 6.00 ± 2.21** | -0.48 ± 1.97 | 1.38 ± 0.29*** | 0.53 ± 0.21* |  |  |
| *M. migrans* | 2.90 ± 2.60 | 0.03 ± 2.28 |  |  | 0.70 ± 0.27* | 2.49 ± 0.36*** |
| *H. pennatus* | -2.16 ± 3.72 | -3.32 ± 2.96 |  |  |  |  |
| *B. buteo* | -2.90 ± 5.80 | 3.76 ± 5.14 | 1.33 ± 1.39 | -2.07 ± 1.57 |  |  |
| *F. tinnunculus + F. naumanii* | 0.36 ± 0.98 | -1.61 ± 1.32 | -5.31 ± 4.71 | 0.51 ± 3.74 |  |  |
| *G. fulvus* | -11.07 ± 3.66** | -9.66 ± 2.46*** |  |  |  |  |
| *A. monachus* | 7.84 ± 6.19 | -0.56 ± 4.46 |  |  |  |  |

Value of the coefficient ± SD of explanatory variables in the analysis of asphalt cells.

Significance levels: * p < 0.05; ** p < 0.01, *** p < 0.001.

adt: Average daily traffic.

^C^ Controlling variable.
